# Viral dynamics and immune responses to foot-and-mouth disease virus in African buffalo (*Syncerus caffer*)

**DOI:** 10.1186/s13567-022-01076-3

**Published:** 2022-08-04

**Authors:** Eva Perez-Martin, Brianna Beechler, Fuquan Zhang, Katherine Scott, Lin-Mari de Klerk-Lorist, Georgina Limon, Brian Dugovich, Simon Gubbins, Arista Botha, Robyn Hetem, Louis van Schalkwyk, Nicholas Juleff, Francois F. Maree, Anna Jolles, Bryan Charleston

**Affiliations:** 1 The Pirbright Institute, Woking, Surrey UK; 2 Carlson College of Veterinary Medicine, Oregon State University, Corvallis, OR USA; 3 UCL Institute of Prion Diseases, London, UK; 4 ARC-OVI Transboundary Animal Disease Section (TAD), Vaccine and Diagnostic Development Programme, Onderstepoort, Gauteng South Africa; 5 State Veterinary Services, P.O. Box 12, Skukuza, 1350 South Africa; 6 Brain Function Research Group, School of Physiology, Faculty of Health Sciences, University of the Witwatersrand, Johannesburg, South Africa; 7 School of Animal, Plant and Environmental Sciences, University of the Witwatersrand, Johannesburg, South Africa; 8 Bill and Melinda Gates Foundation, Seatle, USA; 9 Clinglobal, B03/04 The Tamarin Commercial Hub, Jacaranda Avenue, Tamarin, 90903 Mauritius

**Keywords:** SAT, FMDV, host-viral interaction, innate immune response, acute phase proteins, fever, viral shedding, swab, Carrier, virus isolation

## Abstract

Foot-and-mouth disease (FMD) is one of the most important livestock diseases restricting international trade. While African buffalo (*Syncerus caffer*) act as the main wildlife reservoir, viral and immune response dynamics during FMD virus acute infection have not been described before in this species. We used experimental needle inoculation and contact infections with three Southern African Territories serotypes to assess clinical, virological and immunological dynamics for thirty days post infection. Clinical FMD in the needle inoculated buffalo was mild and characterised by pyrexia. Despite the absence of generalised vesicles, all contact animals were readily infected with their respective serotypes within the first two to nine days after being mixed with needle challenged buffalo. Irrespective of the route of infection or serotype, there were positive associations between the viral loads in blood and the induction of host innate pro-inflammatory cytokines and acute phase proteins. Viral loads in blood and tonsil swabs were tightly correlated during the acute phase of the infection, however, viraemia significantly declined after a peak at four days post-infection (dpi), which correlated with the presence of detectable neutralising antibodies. In contrast, infectious virus was isolated in the tonsil swabs until the last sampling point (30 dpi) in most animals. The pattern of virus detection in serum and tonsil swabs was similar for all three serotypes in the direct challenged and contact challenged animals. We have demonstrated for the first time that African buffalo are indeed systemically affected by FMD virus and clinical FMD in buffalo is characterized by a transient pyrexia. Despite the lack of FMD lesions, infection of African buffalo was characterised by high viral loads in blood and oropharynx, rapid and strong host innate and adaptive immune responses and high transmissibility.

**Supplementary Information:**

The online version contains supplementary material available at 10.1186/s13567-022-01076-3.

## Introduction

Foot and mouth disease (FMD) is an acute vesicular viral disease of domesticated and wild cloven hooved animals characterised as highly contagious with a very short incubation period. In the acute stages of disease in FMD susceptible livestock such as cattle, sheep and pigs, clinical signs include fever, blister-like lesions followed by erosions on the tongue, mouth, snout and feet [1]. FMD is indeed one of the most important livestock farming diseases that is endemic in Africa and causes serious socio-economic impact in the livestock industry and inhibits international trade [2]. Little is known about the pathogenesis of natural FMD infections in wildlife but it has been described that FMD can be fatal in gazelles and warthogs [3] after an experimental infection, while a clinically unapparent infection is typically seen in African buffalo [4, 5].

FMD virus (FMDV) is a small (30 nm in diameter) roughly spherical, non-enveloped, positive-sense single-stranded RNA picornavirus of the genus *Aphthovirus.* Given its serological diversity, FMDV is classified into 7 serotypes: A, O, Asia1 and C (also named Eurasian serotypes) and the Southern African Territories (SAT) 1, 2 and 3 with varying global distribution and causing indistinguishable disease [6].

FMDV is mainly transmitted directly from infected animals in close contact with naïve animals during acute infection. FMDV has a very high rate of transmission and R_0_ values during early stages of the disease were considered to be 21–88 for cattle, 1–14 for sheep [7–9] and very recently, the R_0_ estimated for African buffalo was 5–15.8 [10]. In cattle, the onset of clinical signs occurs 3–4 days after infection and transmission occurs, on average, 0.5 days after the appearance of clinical signs [11] when very high titres of virus are found in the damaged epithelium due to vesicle formation and vesicular fluid [12]. In contrast to cattle, African buffalo develop a sub-clinical infection after being experimentally infected with high doses of the three SAT serotypes, while the same virus strains in young Nguni cattle caused fatal and acute FMD [5]. In cattle, the nasopharyngeal mucosa is the primary site of replication after natural infection, with subsequent dissemination to the lungs followed by viraemia of about 3–5 days of duration [13]. The major mechanism of controlling FMDV infection is the induction of neutralizing antibodies, which are detected as soon as 4 days post-infection (dpi), peak at 14 dpi and are maintained for very long periods of time (years) [1]. The humoral immune response induced by infection or vaccination protects the animal against FMD but does not consistently prevent replication in the nasopharynx and establishment of persistent infection or the carrier status [14].

An innate non-specific immune response based on type I and III interferon (IFN) has been described to play a role in the early protective response against FMDV in pigs and cattle [15–17]. In fact, even though FMDV has developed mechanisms to antagonize the IFN response in vitro [18] type I/III IFN is readily detected in serum after FMDV infection in cattle, pigs, mice and African buffalo [5, 19].

Enhanced production of acute-phase proteins (APPs), haptoglobin and serum amyloid A (SAA) in serumhave been described in cattle during acute infection with FMDV [20]. Interestingly, detection of APP has been used as an indicator of a range of infectious diseases to monitor progression of disease, as a marker to assess animal health and welfare at farms or slaughterhouses, antibiotic treatment efficacy and recently, as a biomarker of other infections in African buffalo [21].

Most African buffalo in sub-Saharan Africa are endemically infected with all three SAT serotypes [22–24] and are considered the main, and for some authors, the sole FMDV reservoir [3] as they may remain persistently infected for many years [25, 26]. Controlling transboundary diseases such as FMD is crucial for improving livestock productivity in endemic regions and allow international trade in livestock products [27]. FMD control in sub-Saharan Africa provides unique challenges because the SAT serotypes are maintained in wildlife and act as a source of infection for livestock [28, 29]. Therefore, an important element of FMD control in livestock in Africa is understanding the pathogenesis and transmissibility in African buffalo. SAT2 is the most widely distributed serotype and is also the serotype most often associated with outbreaks in livestock and wildlife, followed by SAT1 and then SAT3 [30–32]. However, in contrast to cattle, little information is known about the viral dynamics, shedding, transmission rates, and host-immune responses during acute infection in African buffalo.

Therefore, the aim of this study was to fill the knowledge gaps of FMDV infection dynamics and immune-pathogenesis in African buffalo following needle or direct contact infection with three SAT FMDV serotypes. Parameters such as viraemia, viral shedding, clinical outcome and fever, as well as the systemic levels of APP in serum and innate and adaptive immune responses were analysed. Despite the lack of visible clinical signs, infected buffaloes showed high body temperature, high virus titres in blood and nasopharynx, and readily transmitted the virus to naïve buffalo.

## Materials and methods

### Experimental design and sampling

Twenty-four African buffalo (*Syncerus caffer*) were donated by the Hluhluwe-Imfolozi Game Reserve, South Africa; confirmed free from antibodies to FMDV by the OIE Regional Reference Laboratory (ARC-OVI) and transferred to the experimental animal facilities at Skukuza, State Veterinary Services (SVS), Kruger National Park (KNP). Animals were allowed one month for acclimatisation and daily monitoring of their health was performed throughout the experiment. Experimental protocols were approved by the Department of Agriculture, Forestry and Fisheries (DAFF) (sections 20: 12/11/1/8/3/) and the SANParks Animal Ethical Committee (N013-12). Animals were sedated with etorphine hydrochloride and xylazine during experimental procedures and sample collection.

The 24 buffalo, 12 female and 12 male, aging between 10 and 24 months were randomly divided into six groups with two males and two females in each group. Animals in three groups were intradermally challenged with either SAT1, SAT2 or SAT3 FMDV, at a dose of 2.5 × 10^5^ TCID_50_ in the tongue. These groups will be referred to in the manuscript as “needle infected” (NI) animals. Two days after the infection, the remaining three groups of four naïve buffalo were mixed with each of the three inoculated groups; these animals are referred to as “in-contact animals”. FMDV infection dynamics was studied in the buffalo following needle and natural exposure of each of the SAT viruses during the acute phase for 30 days. All the infections were carried out at the same time and the buffalo (4 NI and 4 in-contact per serotype) were kept in outdoor bomas of approximately 300 m^2^ with double fencing separating each boma. Inside each boma, animals were provided with a shed, and food (eragrostis hay and lucerne) and water ad libitum in shared troughs.

Buffalo were monitored for the presence of FMD clinical signs and sampled on days 0 (day of the needle infection), 2 (day when in-contact buffalo are mixed with NI), 4, 6, 8, 11, 14 and 30. Sample collection included blood, oropharyngeal scraping (probang), nasal and tonsil swabs. Whole blood samples collected from the jugular vein were centrifuged to extract serum to measure pro-inflammatory cytokines (type I/III IFN, IFN-γ, TNFα) and APP by ELISA; and specific humoral immune response measurement by virus neutralization test (VNT) and ELISA. Blood samples were also collected in EDTA for leucocyte counts immediately after collection on a Coulter T-890 (Beckman). Serum, probang and tonsil swab samples were collected for the detection of FMDV by RT-qPCR and virus isolation. Probang samples were obtained by gentle erosion of the oropharyngeal epithelium with the probang cup [33]. Epithelium was resuspended in 3 mL of probang buffer (Eagles-hepes supplemented with penicillin/streptomycin, Sigma) and snap frozen in liquid nitrogen. Left and right palatine tonsils were swabbed individually with nylon brushes (Cytotak™ Transwab, Medical Wire), dipped in cryovials containing 0.5 mL of probang buffer and snap frozen in liquid nitrogen [5]. Cotton nasal swabs (Salivette^R^) were soaked in 0.5 mL of PBS and introduced into both nostrils to collect nasal fluid. Swabs were then centrifuged, the liquid collected and aliquoted. All samples were stored at − 80 °C until processing.

### Viruses and cell lines

Virus isolates used for the FMDV challenge were SAT1/KNP/196/91, SAT2/KNP/19/89 and SAT3/KNP/10/90 with accession numbers KR108948, KR108949 and KR108950, respectively. These viruses were originally from buffalo in Kruger National Park, isolated in primary porcine kidney cells (PK) and propagated (5 passages) in IB-RS-2 porcine cell line [5].

IB-RS-2 cells were also used for the virus neutralization assay. ZZR-127 goat epithelial cells were used for virus isolation from tonsil swabs and probang samples and sera [34]. MDBK-t2 (Madin-Darby bovine kidney) cells transfected with a plasmid expressing the human MxA promoter driving a chloramphenicol acetyltransferase (CAT) cDNA were used for the antiviral assay to detect Type I/III IFNs [35]. Cell lines were maintained in minimal essential medium (MEM) supplemented with nutrient F-12 (ZZR-127 cells), hepes, l-glutamine, 10% foetal calf serum and antibiotics (penicillin 100 U/mL and penicillin 100 µg/mL). MDBK-t2 cells were also supplemented with 10 µg/mL of blasticidin (Invitrogen, CA, USA).

### Measurement of abdominal body temperature

Body temperature was measured every five minutes using temperature-sensitive data loggers implanted in all 24 buffalo included in this study plus in 12 other buffalo not exposed to FMDV, as previously described [36, 37]. Experimental protocols were approved by the Animal Research Ethics Committee of the University of the Witwatersrand: 2015/07/31/C. Briefly, after buffalo were sedated, the implantation site was shaved, injected with a local anaesthetic, and disinfected with chlorhexidine gluconate (Hibitane, Zeneca, SA). An incision of about 5 cm was made through the paralumbar fossa. Wax-covered (SASOL wax 1276, SASOL, South Africa) data loggers (DST centi-T, Star-Oddi, Garoabaer, Iceland) were sterilized with an instant sterilant (F10 Sterilant with rust inhibitor, Health and Hygiene (Pty) Ltd, Roodepoort, South Africa) before being placed between the peritoneum and the abdominal muscles and secured to the surrounding muscle with nylon sutures (NY924, size 0, SA). The surgical site was sutured closed with dissolvable sutures (Viamac VM514, size 2) and the surgery wounds coated with an antiseptic spray (Necrospray, Centaur Labs) and an insecticide (0.3% chlorfenvinphos, Swavet, North Riding, South Africa). The sedation was reversedand buffalo were observed until they could stand up. All wounds had healed by the time the experimental procedures started 5 days later.

Adjusted body temperatures from 12 animals that were not exposed to FMDV were plotted from day 0 to 30 of the study (Additional file 1). We used these animals to define normal body temperatures in the buffalo, using the robust method as preferred by the NCCLS guidelines for small sample sizes. This resulted in a reference range of 37–39.5C (± 0.2). However, since animals are represented more than once in the data (time series within an individual) and temperature appears to fluctuate reliably over the day we decided a nonlinear curve fit to the data was a better measure of “normal body temperature” than a static reference range.

We fitted a nonlinear curve to the body temperature data over time to include the effect of nychthemeral changes in body temperature in our detection of fever. We performed an outlier analysis (ROUT Analysis^1^, Q = 10%) but no outliers were found. A nonlinear curve with a sine function was fit to the data using robust nonlinear regression with constraints of amplitude > 0, wavelength = 1, frequency = 1 day and phase shift between 0 and 6.3 (2 pi) (Additional file 1A). The residuals were evaluated (Additional file 1B) and found to vary between − 1.0 and 1.1 °C (Additional file 1C). The best fit values for this line are amplitude = 0.4, wavelength = 1 (constrained), Phaseshift = 3.4, frequency = 1 and baseline temperature = 38.3 °C. We calculated the residuals for the experimentally infected animals using the fitted nonlinear curve with any value above a residual of 1.1, being considered a fever (above ~ 39.3 °C) and omitting any time point within 1 h of a capture period. Data of one animal from SAT1 in-contact group was eliminated due to high readings from day 8 onward—possibly a malfunctioning unit. Using these residuals, we were able to calculate the length of each fever and the time it began (Additional file 2). To calculate the initial day an animal mounted a fever and remove small fluctuations that may not be fever we took the time point at which an animal mounted a fever (first residual above 1.1) and the fever stayed present for at least 6 consecutive hours. For the return to normal body temperature, we had the same requirement, residuals had to be below 1.1 for at least 6 consecutive hours.

### FMDV RNA detection in serum, probang, nasal and tonsil swabs by reverse transcriptase qPCR (RT-qPCR)

RNA templates were extracted from 100 µL of sample (serum, probang, nasal and tonsil swab) to a final elution volume of 80 µL using a MagNA Pure LC RNA isolation kit (Roche, Basel, Switzerland) and the KingsFisher Flex 96 robot (Thermo Fisher Scientific, Waltham, MA, USA). Viral load was determined by means of RT-qPCR using primers targeting the conserved 3D^pol^-coding region of FMDV genome [38]. SAT serotype-specific primers and probes as previously described [5] were also used in tonsil swab samples at day 30 of the study. Forty cycles of PCR were carried out on a Stratagene Mx3005P QPCR system using MXPro MX3005 v3 software (Agilent Stratagene, La Jolla, CA, USA). Cycle threshold (Ct) values were converted to FMDV genome copy number (GCN) by using a linear regression model with serial dilutions of in vitro synthetized RNA standard. Results were expressed as Log_10_ GCN/mL of sample. A cut-off of 1 GCN/5 µL of RNA was used for all samples which resulted in detection thresholds of 2.2 log_10_ FMDV GCN/mL of sample.

### Air sampling

To investigate the possible aerosol transmission of FMDV in buffalo, we collected the aerosols exhaled by the NI buffalo using a Coriolis Air Sampler (Bertin Technologies) [39]. The Coriolis Air Sampler collects the aerosols in a plastic bottle filled with Eagles media with antibiotics that is connected to a high-volume vacuum pump with an airflow rate of 300 L/min. The air sampler was positioned approximately 1 m away from the mouth of the NI buffalo under sedation for 10 min, on days 0, 2, 4, 6 and 8 of the experiment. Aliquots of 1 mL of media collected from the plastic bottle were analysed by RT-qPCR for the detection of viral particles.

### Virus isolation

Virus isolation from the oropharynx (OP) samples (probang and tonsil swab) and serum was performed in a monolayer of ZZR-127 goat epithelial cells following the procedures described by the World Organisation for Animal Health Manual of Diagnostic Test and vaccines for terrestrial Animals, 2022, (Chapter 3.1.8) When no cytopathic effect was observed after 48 h of incubation a second passage of virus was performed on new ZZR-127. Positive cytopathic effects were confirmed for the presence of FMDV by RT-qPCR.

### Detection of FMDV neutralizing antibodies by virus neutralization test (VNT)

Serum samples were assayed for the presence of homologous neutralizing anti-FMDV antibodies by virus neutralization test (VNT) as described elsewhere [40]. Briefly, two-fold dilutions of serum were incubated with 100 TCID_50_ of SAT1, 2 or 3 FMDV in a monolayer of IB-RS-2 cells in 96-well plates for three days. The number of wells with cytopathic effect (CPE) were counted and titres are expressed as the log_10_ of the reciprocal of the highest dilution of serum that neutralized the virus in 50% of the wells. Titres > 1.6 log_10_ are considered to reach the threshold of protection according to Barnett et al. [41].

### Detection of FMDV antibodies against the non-structural proteins by ELISA

Serum samples were analysed for the detection of antibodies against the viral non-structural proteins (NSP). A PrioCHECK FMDV NS ELISA (Prionics®, Lelystad, The Netherlands) was performed according to the manufacturers’ specifications. Results of more than 50% of percentage of inhibition (PI) were considered positive.

### Determination of type I/III IFN, TNF-α and IFN-γ in serum

An Mx/chloramphenicol acetyltransferase (Mx-CAT) reporter assay was used to determine the levels of biologically active IFN in serum samples [42]. Briefly, serum samples were incubated on MDBK-t2 cells for 24 h at 37 °C and 5% CO_2_. Cells were then lysed in lysis buffer and CAT expression, induced by antiviral proteins present in the serum, was determined from the cell lysate using an ELISA kit (catalog number: 113637270001, Roche) according to company instructions. Units of antiviral activity per mL of serum were calculated from a standard curve using recombinant bovine IFN-α [43]. A cut-off of 0.76 IU/mL was established by measuring the average of the basal levels plus twice the standard deviation.

The levels of TNF-α in buffalo sera were determined using a commercial ELISA kit (catalog number: ELB-TNFa-1 RayBiotech, Peachtree Corners, GA, USA) according to the manufacturer’s protocol. Results were expressed as µg/mL of serum. A cut-off of 1.76 µg/mL was established by measuring the average of the basal levels plus twice the standard deviation.

The quantitative determination of IFN-γ in buffalo serum was assayed by a commercial bovine IFN gamma sandwich ELISA test (product code: MCA5638KZZ, Bio-Rad, Watford, UK) following the manufacturer’s specifications. Results are expressed as µg/mL extrapolated from a standard curve of recombinant bovine IFN-γ. A cut-off of 1.04 µg/mL was established by measuring the mean of the basal levels and adding 2 times the standard deviation to the mean value.

### Determination of acute phase proteins in serum: serum amyloid A (SAA) and haptoglobin

Buffalo serum samples were tested for the levels of serum amyloid A (SAA) protein in a sandwich ELISA using the manufacturer’s instructions (cat number: SAA-11; Life Diagnostics, Abu Dhabi, United Arab Emirates). Results were reported as ng/mL. A cut-off of 546 ng/mL was established by measuring the average of the basal levels plus twice the standard deviation.

A commercial kit (cat number: HAPT-11 Life Diagnostics, Inc, West Chester, PA, USA) specific for bovine and based on a sandwich ELISA was used for the quantitative determination of haptoglobin in buffalo serum following the instructions. Results were expressed as ng/mL. A cut-off of 802 ng/mL was established by measuring the average of the basal levels plus twice the standard deviation.

### Statistical analysis

Initial elevation and maximum body temperature, as well as duration of high body temperature was calculated in R (version 3) and analysed by Kruskal–Wallis test. Viral loads in serum and tonsil swab samples, FMDV immune response (VNT, TNF, IFN-γ and type I/III IFN) and acute phase of proteins in serum (Haptoglobin and SAA) over time were analysed by determining, for each animal, the area under the curve (AUC), maximum value and day when the peak value was detected. For virus loads (in serum and tonsil swab), VNT and NSP, first day with a positive value was also identified. Finally, duration of viraemia was estimated for virus loads in serum; duration was defined as the interval between the midpoint of first observation with a log_10_ value and the preceding negative observation and the midpoint of last observation with a log_10_ value and the subsequent negative observation. The response time was measured as time of the first positive value for each parameter minus the first day that FMDV is detected (presence of FMDV in tonsil swab, blood, or nose).

All measurements were compared between NI animals and in-contact animals (regardless of the serotype) and between different serotypes among NI animals and in-contact (corrected by time of exposure) using Kruskal-Wallis test. Median, minimum and maximum values and the Kruskal–Wallis statistics of virus loads, serology and immunological values stratified by serotype (SAT1, SAT2 and SAT3) is shown in Additional file 3. Median, minimum and maximum values and the Kruskal–Wallis statistics of virus loads, serology and immunological values stratified by method of infection (needle versus in-contact) is shown in Additional file 4. Correlation between viral loads in serum and tonsil swabs, initial detection of fever and FMDV in tonsil swabs and serum, were done by Spearman’s rank test.

## Results

### Transmission of FMDV from needle inoculated to in-contact buffalo

FMDV was transmitted readily from NI to all in-contact buffalo within the first week of being mixed. As expected, FMDV was first detected in serum and/or tonsil and nasal swabs in all NI animals synchronous at 2 dpi. FMDV detection in the in-contact buffalo was delayed (*p* < 0.014) compared to NI animals and more variable within and between serotypes, although all in-contact animals were infected within two to nine days of virus exposure. Because of this variation, the analysis of the values of the immunological parameters in the in-contact groups accounted for the day that virus was first detected. FMDV was not detected in any of the air samples collected adjacent to the NI animals after infection (data not shown).

Infection was delayed in one animal in the SAT3 contact group, with FMDV first detected on day 9, and was omitted from analysis due to the limited samples available post onset of infection.

### Clinical signs, body temperatures and leukocyte counts

After FMDV challenge there was no significant change in total white blood cell count (Additional file 5) and only minor mouth lesions were seen in 3 out of 24 animals (two animals from SAT1 NI and one from SAT3 contact groups), at 6–11 dpi. Lesions consisted of small, rounded vesicles of around 4 mm diameter, in the upper dental pad. No lesions were observed in the coronary band or on the tongue, except for the needle tracks where the inoculation occurred.

**Figure 1:**
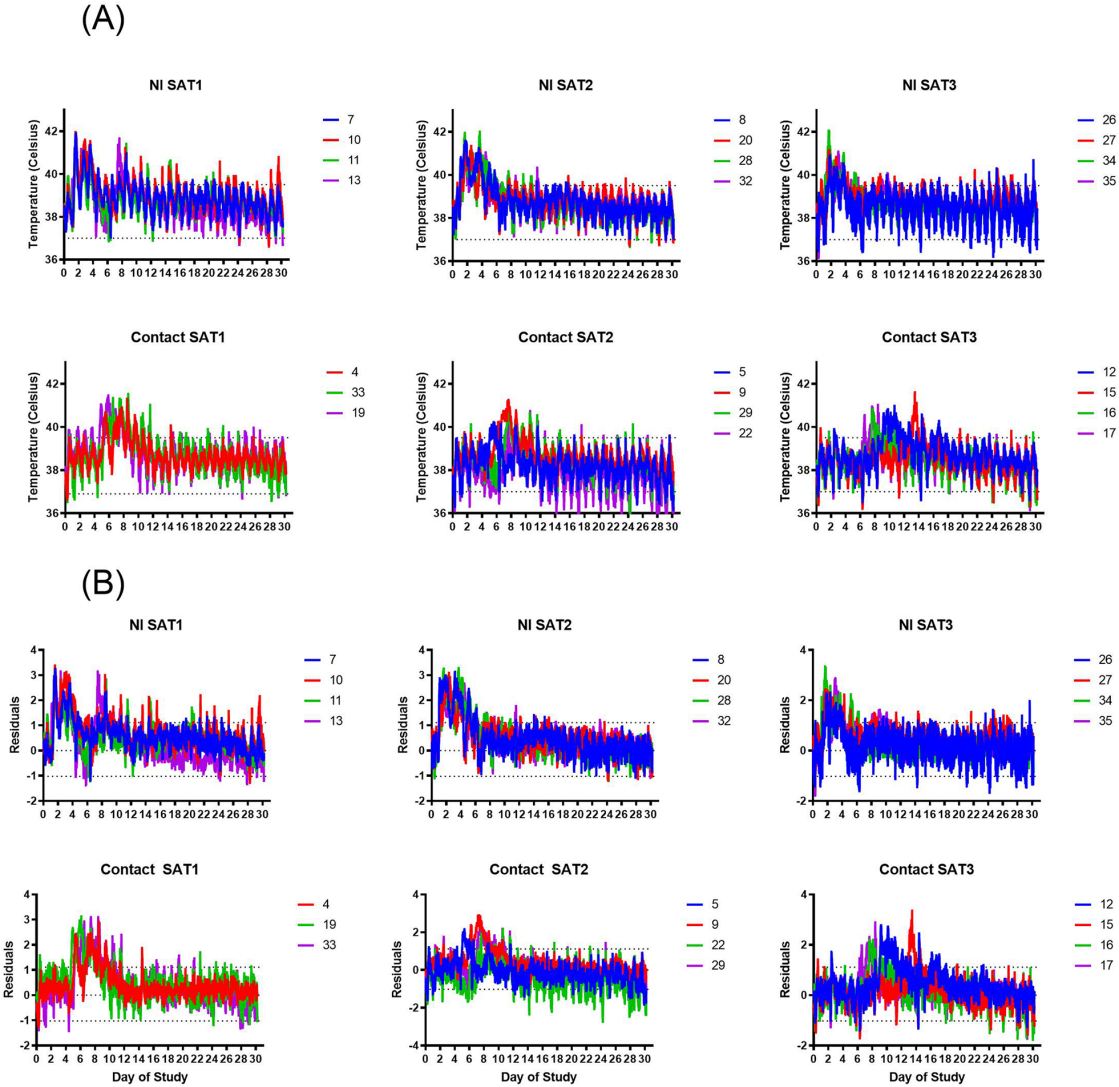
**Body temperature in FMDV infected African buffalo.** Body temperature, measured by temperature-sensitive data loggers, of each individual after needle or in-contact infection with FMDV SAT1 (column 1), SAT2 (column 2) or SAT3 (final column). One animal was eliminated from analysis for SAT 1 in-contact infected due to repeated high readings (animal 2). Panel (**A**) represents the raw temperatures over time. The dotted lines represent the normal temperatures as determined by the calculated reference range. Panel (**B**) represents the residuals from the fitted line, with a value above the dotted line considered a fever if the residuals stay above the line for 6 consecutive hours (72 readings). Body temperature fluctuates during the day and all NI and in-contact buffalo show high temperature within 1–2 days after virus infection (NI) and approximately 5 days after virus exposure (in-contact) and remained elevated for around 3–4 days. SAT2 NI had an earlier initial elevated temperature and compared to SAT1 NI and SAT3 NI (*p* = 0.02) and SAT3 in-contact showed a delay in fever (*p* = 0.006) compared to the SAT2 and SAT3 in-contact. All buffalo from the SAT1 NI and 1 animal from SAT2 and SAT3 showed a short second peak of high temperature at day 8 that lasted approximately 1 day only.

As shown in Figure 1 and Additional file 2, body temperatures were elevated (> 39.3 °C) very soon, approximately 1.2 (± 0.2) days, after needle infection and remained elevated for 3.69 (± 1.17) days, with a peak of maximum temperature of  > 41 °C at 0.8 (± 0.6) days since the start of the fever. The SAT2 NI group showed a quicker response time to initial elevation (1 dpi) compared to 1.3 and 1.4 days for SAT1 and 3 NI, respectively (*p* = 0.01) All animals from SAT1 and one animal from SAT3 NI groups, showed a short second peak of pyrexia at 8 dpi that lasted less than one day. As expected, kinetics of body temperature was less synchronous within and between groups infected by in-contact, reflecting the different time points and virus doses at which the animals were naturally infected from the NI. All contact animals (except one SAT1 buffalo whose temperature device malfunctioned) showed elevated temperatures after 4.9 (± 2.5) days of being in-contact with the NI animals, temperatures peaked around 1.3 (± 0.3) days after the start of the fever and remained elevated for around 3.3 days (± 1.62) days. No statistical differences were observed in the duration, peak temperature or time of peak between in-contact groups except for the day of the initial elevation which was delayed in SAT3 (6.29 days) compared to SAT1 and SAT2 (3.09 days and 3.95 days, respectively) (*p* < 0.006).

### Viral dynamics in blood, nasal swabs and oropharynx

**Figure 2:**
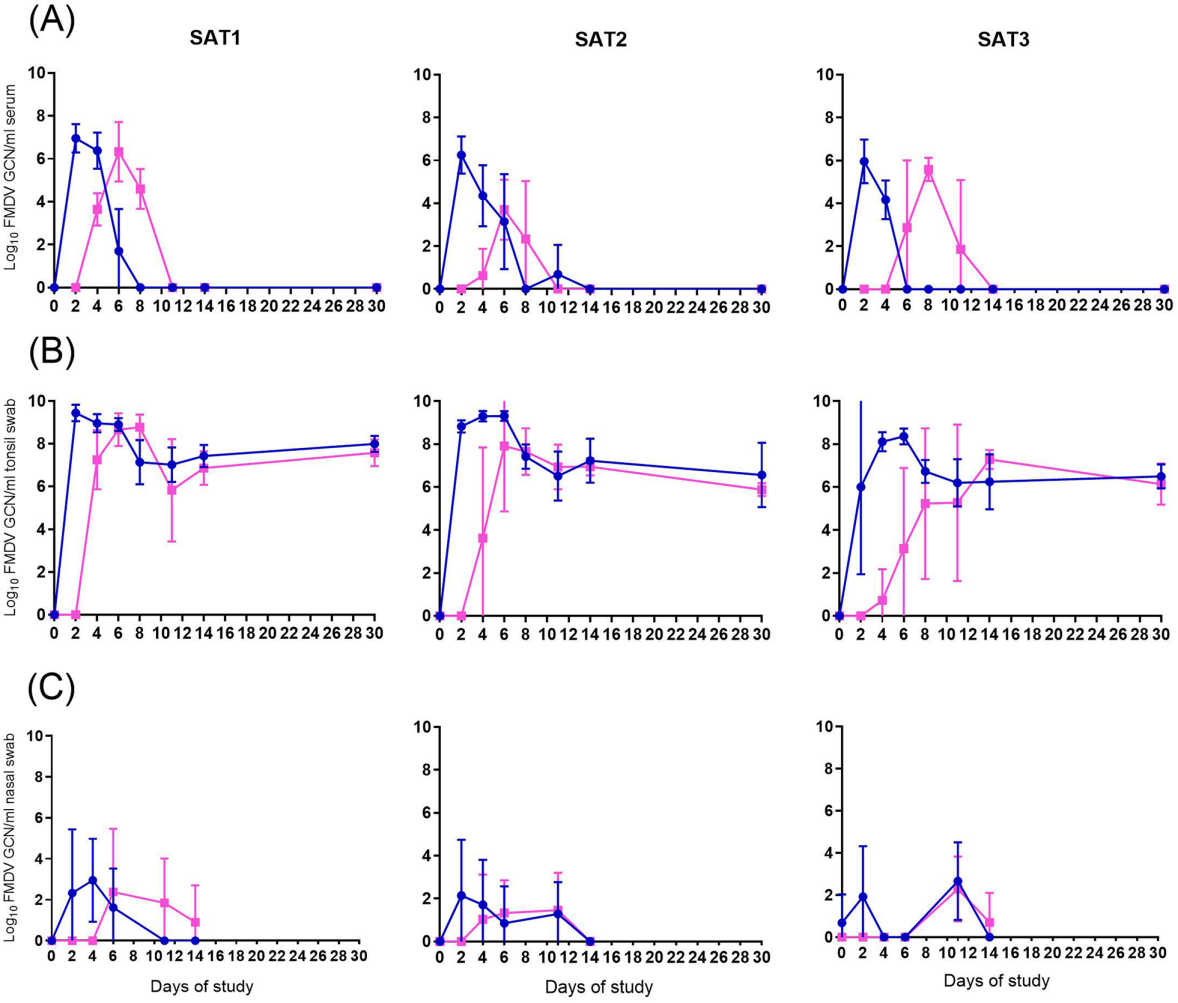
**FMDV genome copy number dynamics in African buffalo.** Detection of FMDV genome copy numbers (GCN) by RT-qPCR in serum (**A**), tonsil swab (solid lines) and probang (dashed lines) (**B**) and nasal swab (**C**) from animals needle infected with SAT1, SAT2 and SAT3 FMDV serotypes (blue lines, round symbol) versus in-contact (pink, square symbol). NI animals were needle infected on day 0 of the study, and in-contact animals were mixed with NI groups on day 2. Graphs represent the mean and SEM for each group at each time point.

Virus genome dynamics in serum samples from NI animals were comparable in all three groups (Figure 2A). The highest FMDV genome copy number was detected by 2 dpi in all the NI animals which coincides with the first detection of virus in blood. Detection of virus genome in blood declined during 4–6 dpi and was undetectable by 8 dpi.

Virus genome dynamics in serum from in-contact animals was more variable within groups and some differences were observed between serotypes. FMDV genome was detected earlier (2 days after virus exposure) in the SAT1 compared to the SAT2 and SAT3 animals (4- and 5-days post virus exposure, respectively, *p* = 0.017), and in general, FMDV detection peaked later, at four days post exposure, compared to NI (*p* < 0.001). The duration of detectable genome in serum was similar irrespective to the route of infection and was found to be longer for the SAT1 group (6.5 days) compared to the SAT2 (4.5 days) and SAT3 (2.5 days) in the in-contact groups (*p* = 0.021). On the contrary, the SAT2 buffalo showed significantly lower viral genome loads in serum with medians of 2.57 GCN/mL versus 3.24 and 3.42 GCN/mL for SAT3 and SAT1, respectively (*p* < 0.028).

FMDV genome could be detected in the oropharynx (OP) 2 days after virus exposure in most of the animals, regardless of the route of infection (Figure 2B). In general, GCN values peaked between 3 to 6 days after virus exposure, except for SAT3 in-contact animals whose peak was delayed until day 5–12 (*p* < 0.045). FMDV genome was detected in tonsil swabs from all buffalo until day 30 of the experiment. NI animals had higher GCN in the OP compared to in-contact infected animals from day 2 to day 30 of the experiment (*p* = 0.004), with the SAT1 NI group showing higher values (5.43 GCN/mL), compared to SAT2 and SAT3 NI groups (5.39 GCN/mL and 5.27 GCN/mL, *p* = 0.048). By 30 dpi, tonsil swabs were analysed by RT-qPCR using SAT specific primers and results indicated that no evidence of cross-infection was detected in any of the groups housed separately during the experiment (Additional file 6).

The initial detection of FMDV genome in serum correlated with the initial detection of virus in tonsil swabs (r^2^ = 0.84) although it was 2 days delayed in 3 out of 12 animals. Similarly, the first day of detection of virus in blood and tonsil swab correlated with the initial elevation of temperature (r^2^ = 0.96 and 0.84, respectively), although fever could be detected 1.17 (± 1.3) days after the detection of virus shedding and 0.7 (± 0.7) days after the presence of virus in blood (Additional file 7).

Virus genome was first detected in nasal swabs on 2 and 6.5 days post virus exposure (group mean values) in NI and in-contact groups, respectively (Figure 2C). Most of the animals were negative by 14 dpi. Contrary to the high GCN in blood and OP, virus genome detection in nasal swabs was intermittent and reached maximum values of 3.4 and 3.34 GCN/mL in NI and in-contact groups, respectively. No statistical differences in the dynamics of shedding in nasal swabs were observed between groups.

Serum, probang, and tonsil and nasal swabs were also analysed by virus isolation (VI) from 2–30 days of the study. On day 2 and 4 after virus exposure, only 66% and 17% of the serum samples, respectively, were positive for virus isolation which contrasts with the high RT-qPCR values in all serum samples on these days. Virus was isolated from 87 and 71% of the RT-qPCR positive samples taken at days 8, 14 and 30 from tonsil swab and probang, respectively. Also, the mean GCN from all VI positive samples was higher in tonsil swabs (*p* < 0.001) (Additional file 8), indicating that a tonsil swab is the most reliable sample for detecting FMD live virus and genome in African buffalo. On day 30 of the experiment, infectious virus was isolated from tonsil swabs and/or probang from 16 (9 NI and 7 in-contact), out of 24 (66.6%) infected animals (Table 1), however there was no association between the route of virus exposure with the carrier status (*p* = 0.553, respectively). Infectious virus could not be isolated from any nasal secretions.

**Table 1:** Number of animals with vesicles and number of animals that were carrier by day 30 of the study

	NI				Contacts				*p*-value
	SAT1	SAT2	SAT3	Total NI (%)	SAT1	SAT2	SAT3	TOTAL contacts (%)	
Clinical signs (vesicles)	2	0	0	2 (16.7)	0	0	1	1 (8.3)	1
Carrier at day 30 (VI+)	4	2	3	9 (75)	3	1	3	7 (58.3)	0.556

### Humoral immune response to FMDV

**Figure 3:**
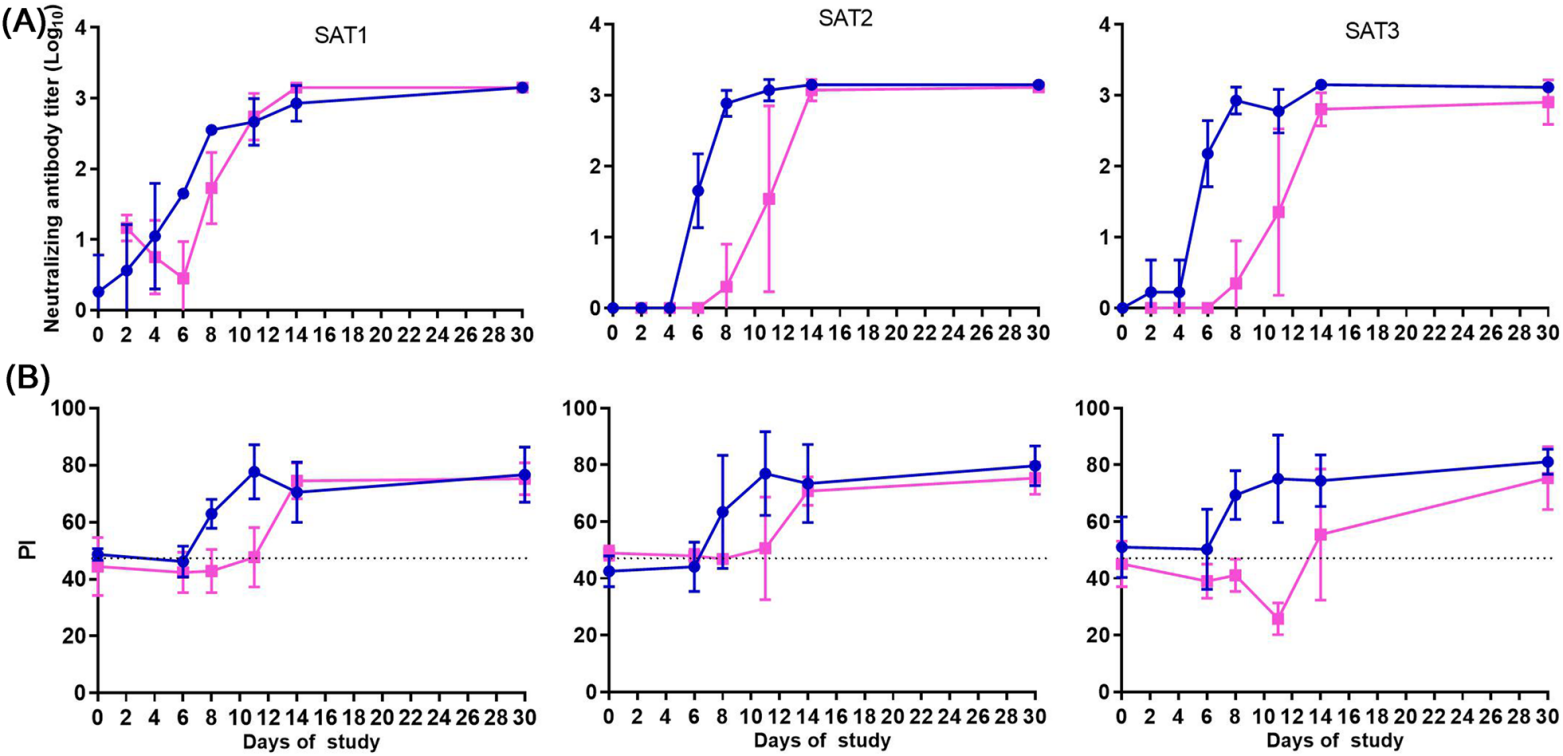
**Specific humoral immune response induced by FMDV infection.** Neutralizing antibody titers in log_10_ (**A**) and presence of antibody levels against FMDV non-structural proteins (NSP) (**B**), induced by SAT1, SAT2 and SAT3 FMDV infected animals by needle infection (blue lines, round symbol) or by contact challenge (pink, square symbol). NSP results are expressed by percentage of inhibition (PI) and cut-off is determined at 50% (dash line). Graphs represent the mean and SEM for each group at each time point.

The specific humoral immune response induced by the different FMDV SAT serotypes after NI were not significantly different, however differences were observed in in-contact groups (Figure 3A). In general, FMDV infected buffalo developed virus neutralizing antibody titres (VNTs) within 2 to 6 days post-infection. VNTs rapidly increased after first detection and were maintained at their maximum titres until the end of the study on day 30. The route of infection did not influence the magnitude of the VNTs, but NI animals reached protective titres faster compared to in-contact animals (*p* < 0.002) and among the in-contact animals, the onset of the response was faster in SAT1 group (*p* < 0.021), showing comparable levels with NI animals.

Antibodies against the non-structural proteins (NSP) of FMDV were first detected at 8 dpi for the NI groups and significantly delayed in the in-contact groups (12 dpi, *p* = 0.001). The NSP antibody titres remained consistently elevated until day 30 of the study (Figure 3B).

### Levels of Haptoglobin and SAA in serum of FMDV infected buffalo

**Figure 4:**
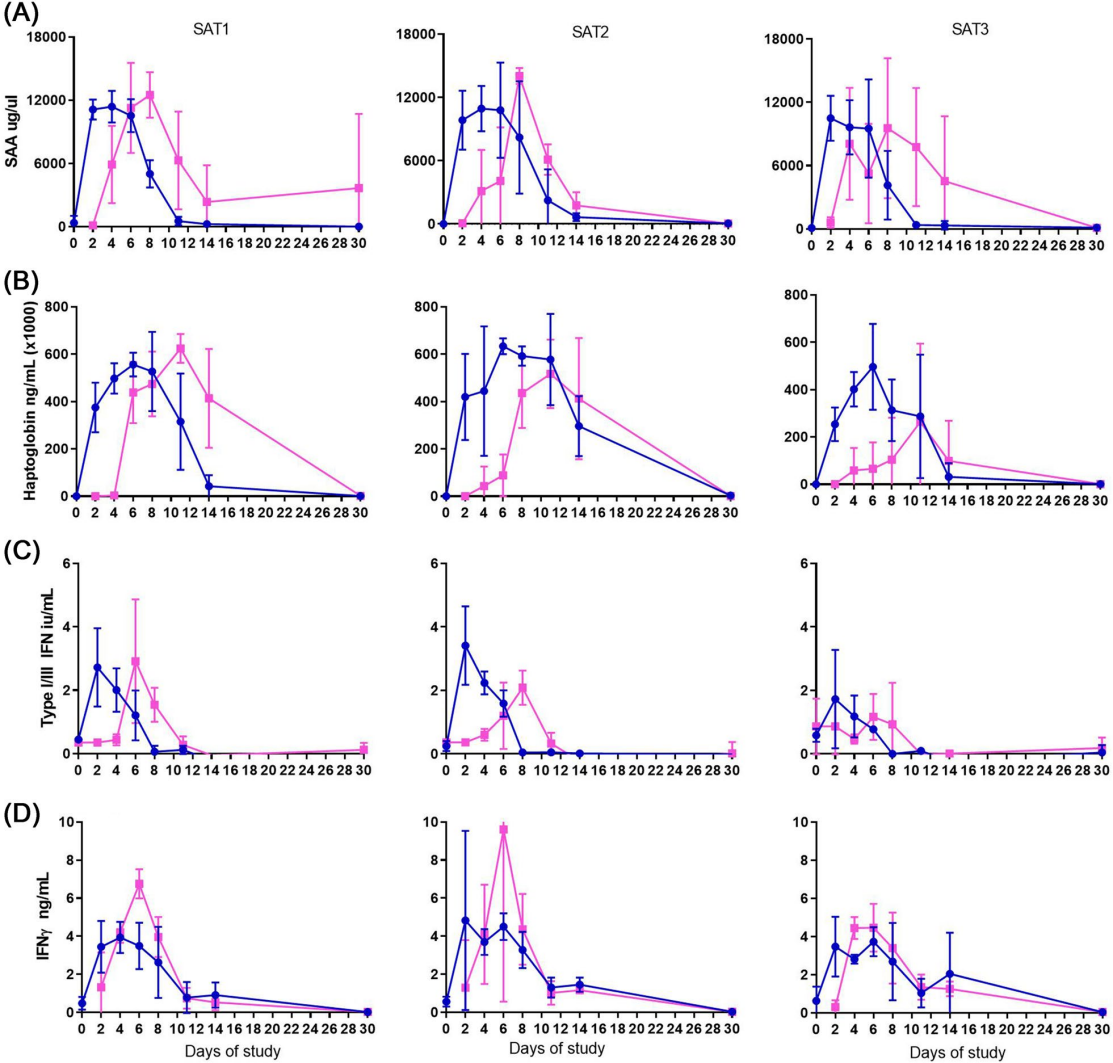
**Innate immune response and acute phase proteins induced by FMDV in African buffalo.** Concentrations of SAA (**A**), hatoglobin (**B**), type I/III IFN (**C**) and IFNɣ (**D**) in serum from animals infected with SAT1, SAT2 or SAT3 FMDV serotypes by NI (blue lines, round symbol) or contact challenge (pink lines, square symbol). Graphs represent the mean and SEM for each group at each time point. Cut-offs for SAA and hatoglobin were established at 546 and 802 ng/µL and cut off for type I IFN and IFNɣ was established at 0.76 iu/mL and 1.04 ng/mL, respectively.

Serum amyloid A (SAA) and haptoglobin were detected during acute FMD infection in buffalo. High concentrations of SAA were detected in serum of all buffalo immediately after viral infection (Figure 4A). Serum concentrations rapidly increased and peaked by 4–6 dpi. Levels declined progressively after the peak and by 14 dpi SAA levels were undetectable. While the total SAA response was not different across serotypes and route of infection, the induction of SAAs was delayed in in-contact animals (*p* < 0.004), however, the duration of SAA in blood and their peak levels were higher compared to NI animals (*p* < 0007).

Similar dynamics were observed in the concentration of haptoglobin in serum (Figure 4B) although levels were maintained for longer than SAA; by day 30 of the study, haptoglobin levels in all animals were normal. No differences in haptoglobin in serum were observed among the different routes of infection, but the magnitude of the response was the highest within the SAT2 NI groups (*p* < 0.018).

### Innate immune response induced in FMDV infected buffalo

The dynamics of Type I/III IFNs and IFN-γ in serum were very similar in response to all SAT infections (Figure 4C). Type I/III IFN responses were detected in the serum readily after infection (2 dpi) for approximately 6 days in most of the animals, but the maximum levels were reached later in in-contact groups (6 dpi versus 2 dpi) (*p* = 0.013). Similarly, the induction of IFN-γ was detected at 2 dpi and peaked at 6 dpi with maximum values for in-contact animals higher than NI (*p* = 0.036) (Figure 4D). No differences in dynamics of Type I/III IFN and IFN-γ antiviral cytokines were found across serotypes, however some animals from the in-contact group had detectable levels to these cytokines even before FMDV was detected (*p* = 0.031). TNF-α could not be consistently detected in FMDV infected buffalo (data not shown).

## Discussion

This study represents the most complete characterization of viral dynamics and immune responses to FMDV infection in African buffalo. FMD in African buffalo is generally regarded as mild or asymptomatic, since no (or very few) vesicles are observed even after a high dose of FMDV challenge [5, 44, 45]. Consistent with these previous reports, small vesicles restricted to the dental pad were only observed in two SAT1 NI buffaloes and one SAT3 in-contact challenged buffalo, contrary to cattle that present with vesicles at multiple sites, generally on the feet and tongue, after the onset of fever [1]. However, using temperature loggers, we demonstrated for the first time that African buffalo are indeed systemically affected by FMDV and develop consistent pyrexic responses early after needle infection (average of 1.26 days) that last, on average, 3.34 days. In general, in-contact animals showed increased body temperature within the first week (in average 4.9 days) of being in contact with the NI buffalo. The initial elevation of temperature of in-contact animals correlated with the first day that FMDV genome was detected in serum and in oropharynx, although detection of FMDV genome in oropharynx and serum preceded by 1.17 (± 1.3) days and 0.7 (± 0.7) days, respectively, the detection of fever. These results indicate that although detection of fever in-contact groups does not allow for a more sensitive detection time of transmission, it is a good correlate of transmission during FMDV infection in buffalo, similar to cattle [12]. In fact, in the absence of FMD lesions, body temperature could probably be the most important correlate of transmission in African buffalo. Interestingly, SAT2 NI showed an earlier increased temperature compared to SAT1 and SAT3 infected animals, however this increased temperature was not associated with higher virus loads in serum or virus replication in the oropharynx. Also, the second peak of fever detected in all the SAT1 NI and one SAT2 NI and SAT3 NI couldn’t be associated with any detection of virus in blood. Within one week of FMDV exposure, buffalo also showed high levels of SAA in serum, similar to the profile detected in cattle [20]. Interestingly, SAT2 NI animals showed higher levels of haptoglobin in serum (*p* < 0.018) compared to SAT1 and SAT3 challenged animals. Acute phase proteins are non-specific markers of inflammation, and although most buffalo did not show FMD lesions, they were all systemically affected by virus infection. Stress associated with handling and sedation has been described to induce high levels of APPs in serum [46]. However, this was ruled out since the buffalo in our study were sedated at different time points along the experiment, and we only observed a positive association with APPs when high levels of virus was detected in the blood and tonsil swabs, indicating that these proteins could be used as a surrogate marker of FMDV infection in African buffalo, as previously suggested [21].

It has been described that FMDV in cattle is highly contagious and R_0_ have been estimated to be between 21 and 88, [7, 9] even though the infectious period is brief (1.7 (0.3–4.8) days) [12]. Moreover, in domestic cattle there is a positive association between transmission, and the presence of the virus in air, and the onset of FMD clinical signs [11]. In this study, despite the lack of vesicles, and the absence of virus in air samples, and nasal swabs, all contact buffalo were readily infected after being in contact with the NI animals. These findings suggest that close-contact transmission of the SATs between buffalo, might be more efficient than transmission by aerosol. Further work is needed to investigate the detection limits of the air sampling protocol in measuring emissions from acutely infected buffalo (e.g., day to day variation and time of exposure) [47] and study which form of transmission is most seen between individuals of this gregarious species.

Although detection of FMDV in nasal swabs was low, high levels of virus and virus genome were detected in the palatine tonsil swabs during the first 4–6 days after infection in all NI animals. These results indicate that the tonsils might be the main source of infectious virus in buffalo rather than vesicular lesions as described for cattle [12]. When comparing both types of pharyngeal samples, higher viral genome copies were detected by qRT-PCR in tonsil swabs compared to probang (*p* < 0.001); these results corroborate previous findings suggesting that tonsil swabs performed better than probang for FMDV diagnosis [5]. Viral loads in tonsil swabs decreased over time; however, 66% of the buffalo were still shedding virus by day 30 of the experiment.

FMDV was first detected concomitantly in tonsil swabs and blood from most of the animals within the first week after FMDV infection, only three animals from the in-contact group showed earlier detection in tonsil swabs than blood. In contrast, one animal from the NI group showed FMDV in blood before tonsil swab. It has been reported in cattle that virus detected in oropharynx provides the earliest indication of infection; but virus in the blood and nasal fluid may also be good candidates for preclinical indicators of infectiousness when virus levels exceed certain thresholds [11]. In this study, the presence of virus genome in nasal swabs was not easily detected and not consistent within groups (5 out of 24 animals were negative at all time points).

Virus genome could be detected in blood from infected buffalo for approximately 4 to 6 dpi, which is a longer duration than measured in cattle (2–4 dpi) [1, 12, 48]. Viral genome in blood correlated closely with the detection of viral genome in tonsil swabs until the appearance of neutralizing antibodies. Soon after neutralizing antibodies were detected, the virus was cleared completely from the bloodstream, around 6 dpi. Similar to cattle, FMDV detection in the oropharynx or tonsil swab was not affected by the presence of neutralizing antibodies [14]. High FMDV genome copy numbers were maintained in palatine tonsil until late after infection when the titres of neutralizing antibodies were maximum. In fact, by day 30 of the study, FMDV could be isolated from tonsil swabs and/or probang in 16 out of 24 animals and these were identified as carriers. We could not find any association of the carrier state with the route of infection or acute host responses, as suggested for cattle [20]. We and others have demonstrated that buffalo can remain persistently infected with FMDV for months and years [5, 25, 49] and although transmission from carrier buffalo to naïve is difficult to reproduce [44, 50], a recent publication demonstrated that it is indeed the inclusion of occasional transmission from carriers that rescues FMDV from extinction in isolated African buffalo populations [10].

Altogether, these results demonstrated similar dynamics of FMDV infection and immune responses after needle infection or direct in-contact challenge in African buffaloes compared to cattle, despite marked differences in the clinical outcome [5, 20]. Also, similar transmission, viral dynamics and neutralizing antibody responses have been described for FMDV infected water buffalo (*Bubalus bubalis*), a domestic buffalo commonly farmed in Asia; however, water buffalo showed foot and mouth vesicles, like cattle [51–53]. The reasons for the different clinical outcomes between the host species remains unclear, in addition to the lack of understanding of the mechanisms responsible for the tissue distribution of FMD vesicles in cattle [54]. We have demonstrated for the first time that pyrexia is a consistent clinical sign of FMD in African buffalo. In general, needle challenge leads to a synchronous, faster and higher viral loads in blood and oropharynx and specific humoral immune response while the innate and acute immune responses were similar in needle and in-contact challenged buffaloes. These differences could be explained by the variable time and the lower dose of infection in the in-contact group compared to high doses of virus in NI. The SAT1 virus was detected more rapidly after challenge compared to the SAT2 and SAT3 viruses and transmitted more readily to naïve buffalo. These results agree with our previous studies where we showed during mixed infections in individual buffalo, over time SAT1 persisted for longer periods compared to SAT2 and SAT3 viruses [5, 55]. The results are also consistent with our observation during a long-term study of an isolated buffalo herd that demonstrated SAT1 viruses persist more readily in a population [10]

These data provide important information to help understand the marked clinical differences between cattle and African buffalo in their response to FMDV infection. We have demonstrated that the typically mild clinical signs in African buffalo are not because virus replication or shedding are controlled and are not associated with a suppressed immune response to FMDV. We have also demonstrated that naïve buffaloes kept in contact with acutely infected buffaloes are readily infected despite the absence of high titre virus in vesicular fluids or lesions. Further studies are required to investigate cell mediated immune responses, and to determine if this arm of the immune response is accountable for the markedly different clinical outcomes in African buffalo compared to cattle. These data form a foundation for modelling the interplay of viral and immune response dynamics within African buffalo host and understanding the pathogenesis of these highly contagious viruses in populations of their natural reservoir host.

## Supplementary Information

**Additional file 1. Normal body temperature in African buffalo. A** The black line is the fitted nonlinear curve, while the green points represent the data from 12 animals, with temperatures collected every 5 min. **B** Residuals over time from the nonlinear regression in A. **C** A scatter plot showing the range of residuals which were found to vary between −1.022 and 1.114, which we assume is normal physiological variation.

**Additional file 2. Calculations of the residuals of body temperature for all African buffalo infected with SAT1, SAT2 and SAT3 over time; and its median values (minimum–maximum) and the Kruskal–Wallis statistics stratified by serotype.**

**Additional file 3. Median values (minimum–maximum) and the Kruskal–Wallis statistics of virus load, serology and hematology values stratified by serotype (SAT1, SAT2 and SAT3).**

**Additional file 4. Median values (minimum–maximum) and the Kruskal–Wallis statistics of virus load, serology and hematology values stratified by method of infection (needle infected vs contact).**

**Additional file 5. White blood cell (WBC) count from all NI and contact animals along the study.**

**Additional file 6. RT-qPCR data expressed as Log**_**10**_** FMDV GCN/mL from tonsil swabs at day 30 of the study using the 3D and SAT specific primers and probe.**

**Additional file 7. Dynamics of body temperature versus viral dynamics. A** Comparison of timeframes in which parameters such as elevated temperature (temp), FMDV presence in tonsil swabs and serum appear in the course of the infection after needle infection (NI) and in-contact exposure of FMDV (12 animals per group). **B** Duration of the temperature and presence of FMDV in serum in NI and in-contact groups. Bars indicate the median of the groups.

**Additional file 8. Boxplots showing PCT values stratified by virus isolation category (negative/positive) in probang and tonsil swab.**

## Data Availability

The datasets analyzed during the current study are available from the corresponding authors upon request.

## Abbreviations

APP: acute phase proteins
Co: contact infected
dpi: days post infection
FMD: foot and mouth disease
FMDV: foot and mouth disease virus
GCN: genome copy number
IFN: interferon
KNP: Kruger National Park
MAbs: monoclonal antibodies
NI: needle infected
NSP: non-structural protein
OP: oropharynx
PI: percentage of inhibition
SAA: serum amyloid A
SAT: Southern Africa Territories
TNF: tumor necrosis factor
VNT: virus neutralizing test
