# Supplementary figures and images for: Viral dynamics and immune responses to foot-and-mouth disease virus in African buffalo (Syncerus caffer)
Source: Vet Res. 2022 Aug 4;53:63. doi: 10.1186/s13567-022-01076-3 (PMC9351118; doi:10.1186/s13567-022-01076-3)

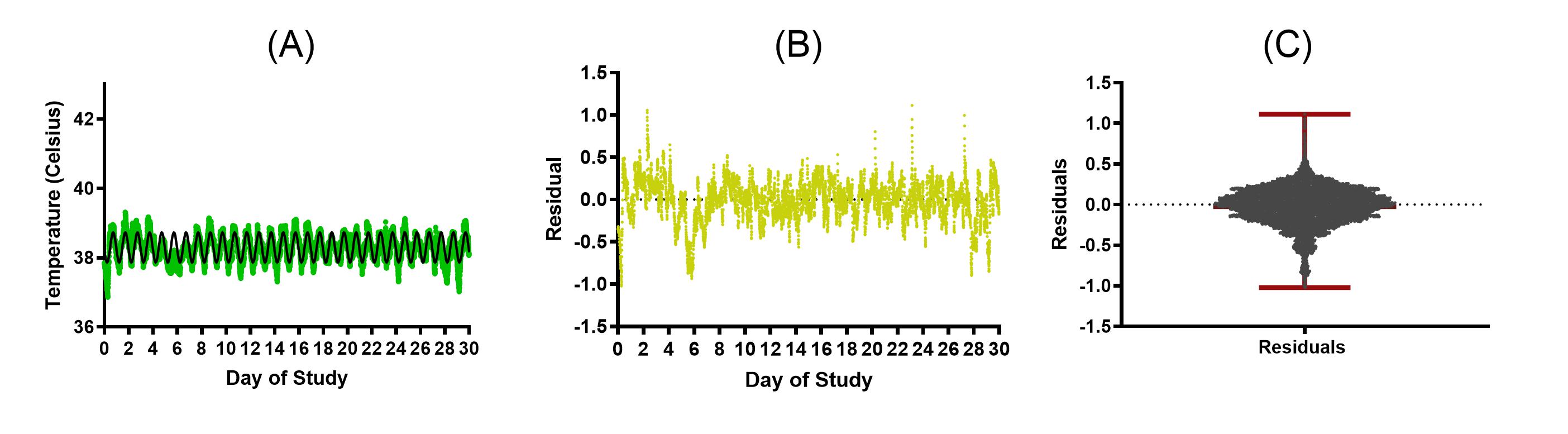

Supplement: Supplementary file 1 — Additional file 1. Normal body temperature in African buffalo. A The black line is the fitted nonlinear curve, while the green points represent the data from 12 animals, with temperatures collected every 5 min. B Residuals over time from the nonlinear regression in A. C A scatter plot showing the range of residuals which were found to vary between −1.022 and 1.114, which we assume is normal physiological variation. [file 13567_2022_1076_MOESM1_ESM.jpg]

(A) (B)


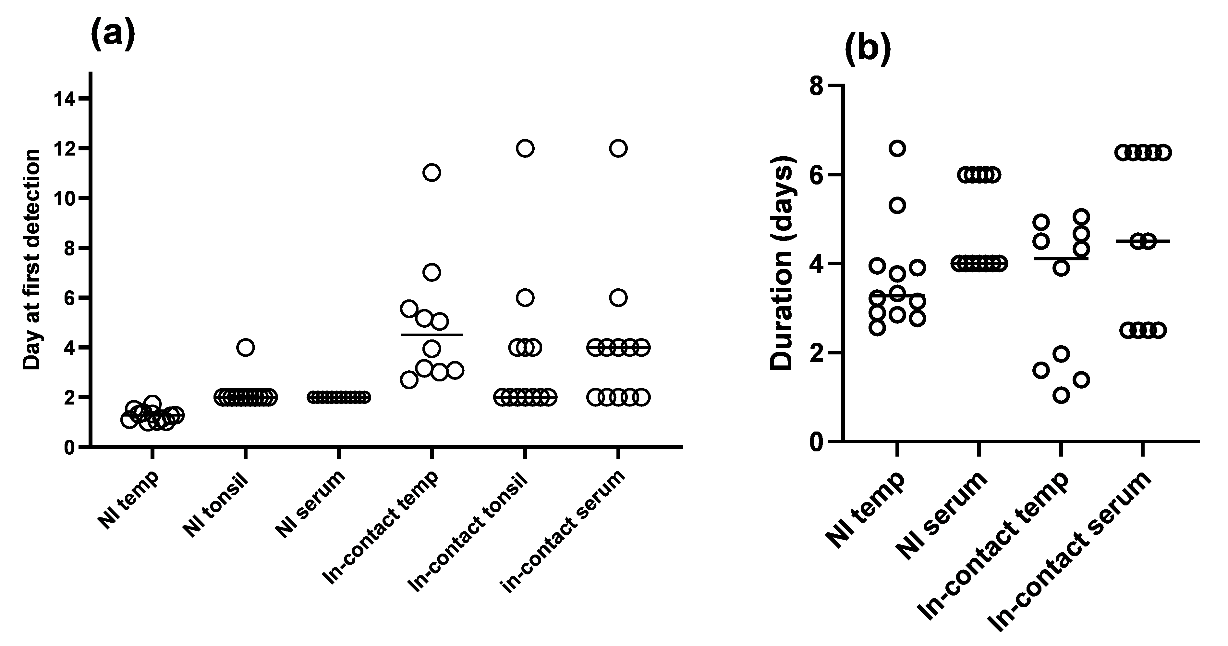

Supplement: Supplementary file 7 — Additional file 7. Dynamics of body temperature versus viral dynamics. A Comparison of timeframes in which parameters such as elevated temperature (temp), FMDV presence in tonsil swabs and serum appear in the course of the infection after needle infection (NI) and in-contact exposure of FMDV (12 animals per group). B Duration of the temperature and presence of FMDV in serum in NI and in-contact groups. Bars indicate the median of the groups. [file 13567_2022_1076_MOESM7_ESM.docx]

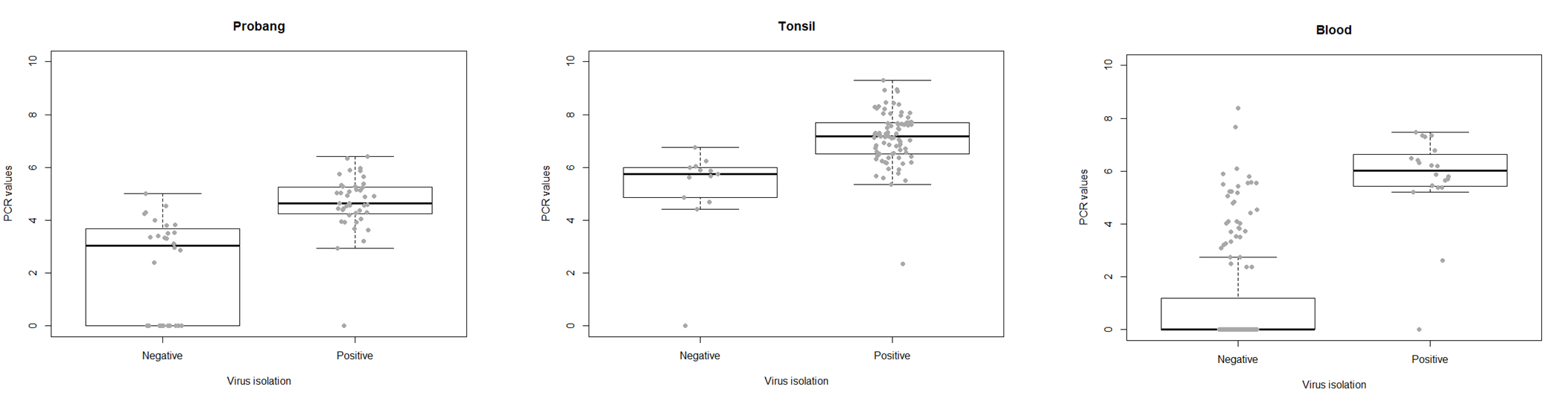

Supplement: Supplementary file 8 — Additional file 8. Boxplots showing PCT values stratified by virus isolation category (negative/positive) in probang and tonsil swab. [file 13567_2022_1076_MOESM8_ESM.docx]
