# Supplementary material for: Viral dynamics and immune responses to foot-and-mouth disease virus in African buffalo (Syncerus caffer)
Source: Vet Res. 2022 Aug 4;53:63. doi: 10.1186/s13567-022-01076-3 (PMC9351118; doi:10.1186/s13567-022-01076-3)
Supplement: Supplementary file 2 — Additional file 2. Calculations of the residuals of body temperature for all African buffalo infected with SAT1, SAT2 and SAT3 over time; and its median values (minimum–maximum) and the Kruskal–Wallis statistics stratified by serotype. [file 13567_2022_1076_MOESM2_ESM.docx]

| **Group** | **Animal ID** | **Max Temp (peak)** | **Time of peak (days)** | **Time of Initial Elevation (days)** | **Length of Fever in Days** | **Time of 2^nd^ peak (days)** | **Length of Fever 2^nd^ peak** |
| --- | --- | --- | --- | --- | --- | --- | --- |
| **SAT1 NI** | 7 | 41.88 | 1.61 | 1.33 | 3.77 | 8.38 | 0.39 |
| **SAT1 NI** | 10 | 41.96 | 1.58 | 1.32 | 3.91 | 8.39 | 0.26 |
| **SAT1 NI** | 11 | 40.91 | 2.39 | 1.39 | 2.77 | 8.41 | 0.35 |
| **SAT1 NI** | 13 | 41.98 | 1.65 | 1.26 | 2.9 | 7.02 | 2.61 |
| **Median values (min-max)** | | 41.92 (40.91-41.98) | 1.63 (1.58-1.65) | 1.32 (1.26-1.39) | 3.34 (2.77-3.91) | 8.05 (70.2-8.41) | 0.9 (0.26-2.61) |
| **SAT2 NI** | 8 | 41.04 | 3.18 | 1 | 3.33 | 4.94 | 0.42 |
| **SAT2 NI** | 20 | 41.11 | 2.36 | 1.1 | 3.22 | NA | NA |
| **SAT2 NI** | 28 | 42 | 1.66 | 1.02 | 5.31 | NA | NA |
| **SAT2 NI** | 32 | 41.26 | 1.56 | 1.03 | 6.59 | NA | NA |
| **Median values (min-max)** | | 41.19 (41.04-42) | 2.1 (1.59-3.18) | 1.03 (1-1.1) | 4.32 (3.22-6.59) |  |  |
| **SAT3 NI** | 26 | 40.43 | 2.39 | 1.73 | 2.56 | NA | NA |
| **SAT3 NI** | 27 | 41.08 | 1.74 | 1.52 | 2.85 | NA | NA |
| **SAT3 NI** | 34 | 42.05 | 1.67 | 1.14 | 3.15 | 4.95 | 0.53 |
| **SAT3 NI** | 35 | 40.89 | 3.08 | 1.29 | 3.95 | NA | NA |
| **Median values (min-max)** | | 40.99 (40.55-41.81) | 1.79 (1.7-2.99) | 1.41 (1.14-1.73) | 3.0 (2.56-3.95) |  |  |
| **NI *P*-value** | | 0.63 | 0.1 | **0.01** | 0.24 | NA | NA |
| **SAT1 contact** | 4 | 41.34 | 8.5 | 5.09 | 4.93 | NA | NA |
| **SAT1 contact** | 19 | 41.07 | 6.11 | 4.7 | 5.05 | NA | NA |
| **SAT1 contact** | 33 | 41.14 | 7.44 | 5.17 | 4.5 | NA | NA |
| **Median values (min-max)** | | 41.14 (41.07-41.34) | 7.44 (6.11-8.5) | 5.09 (4.7-5.17) | 4.93 (4.5-5.05) |  |  |
| **SAT2 contact** | 5 | 40.09 | 5.31 | 5.01 | 1.6 | NA | NA |
| **SAT2 contact** | 9 | 40.83 | 7.32 | 5.95 | 4.67 | NA | NA |
| **SAT2 contact** | 22 | 40.74 | 10.57 | No sustained | No sustained | NA | NA |
| **SAT2 contact** | 29 | 40.72 | 7.52 | 7.05 | 3.9 | NA | NA |
| **Median values (min-max)** | | 40.73 (40.25-40.83) | 7.44 (6.11-8.5) | 5.95 (5.01-7.05) | 3.39 (1.6-4.67) |  |  |
| **SAT3 contact** | 12 | 40.6 | 9.22 | 9.02 | 4.33 | NA | NA |
| **SAT3 contact** | 15 | 41.61 | 13.45 | 13.03 | 1.97 | NA | NA |
| **SAT3 contact** | 16 | 40.15 | 8.12 | 7.56 | 1.04 | NA | NA |
| **SAT3 contact** | 17 | 41.08 | 8.41 | 7.18 | 1.39 | NA | NA |
| **Median values (min-max)** | | 40.84 (40.26-41.61) | 8.81 (8.12-13.45) | 8.29 (7.18-13.03) | 1.68 (1.04-4.33) | NA | NA |
| **Contact *P*-value** | | 0.2 | 0.26 | **0.006** | 0.07 |  |  |
