# Supplementary material for: Viral dynamics and immune responses to foot-and-mouth disease virus in African buffalo (Syncerus caffer)
Source: Vet Res. 2022 Aug 4;53:63. doi: 10.1186/s13567-022-01076-3 (PMC9351118; doi:10.1186/s13567-022-01076-3)
Supplement: Supplementary file 3 — Additional file 3. Median values (minimum–maximum) and the Kruskal–Wallis statistics of virus load, serology and hematology values stratified by serotype (SAT1, SAT2 and SAT3). [file 13567_2022_1076_MOESM3_ESM.docx]

| **Parameter** | **Needle infected - Median (min-max)** | | | **P value** | **Contact - Median (min-max)** | | | **P value** |
| --- | --- | --- | --- | --- | --- | --- | --- | --- |
|  | **Sat1** | **Sat2** | **Sat3** |  | **Sat1** | **Sat2** | **Sat3** |  |
| ***VIROLOGY***  **Virus load in serum**  •AUC (log_10_)  •Day viremia starts  •Day viremia peaks  •Peak value  •Duration (days)  **Virus load in tonsils**  •AUC (log_10_)  •First day detected  •Day peaks  •Peak value  **Nasal swab**  •First day detected  •Day peaks  •Peak value  **Day virus first detected** | 3.37 (3.19-3.61)  2 (2-2)  2 (2-4)  7.07 (6.31-7.67)  5 (4-6)  5.43 (5.36-5.49)  2 (2-2)  3 (2-4)  9.54 (9.12-9.98)  3 (2-6)  4 (2-6)  3.96 (2.78-6.57)  2 (2-2) | 3.48 (3.02-3.53)  2 (2-2)  2 (2-2)  6.25 (5.20-7.31)  6 (4-6)  5.39 (5.26-5.42)  2 (2-2)  5 (4-6)  9.43 (9.32-9.63)  6.5 (2-11)  6.5 (2-11)  2.95 (2.53-5.29)  2 (2-2) | 3.01 (2.92-3.08)  2 (2-2)  2 (2-2)  5.58 (5.22-7.46)  4 (4-4)  5.27 (5.11-5.36)  2 (2-4)  6 (4-6)  8.67 (7.99-9.70)  2 (2-11)  11 (2 -11)  3.83 (0-4.95)  2 (2-2) | 0.058  -  0.368  0.232  0.111  **0.048**  0.368  0.050  0.232  0.820  0.698  0.618  - | 3.42 (3.36-3.53)  2 (2-2)  4 (4-4)  5.71 (5.51-8.39)  6.5 (6.5-6.5)  5.27 (4.81-5.37)  2 (2 - 2)  6 (4-6)  9.13 (7.93-9.63)  4 (4-4)  6.5 (4-9)  5.33 (4.20-6.47)  2 (2-2) | 2.57 (2.00-3.15)  4 (2-4)  4 (4-6)  4.13 (2.50-5.64)  4.5 (2.5-6.5)  5.16 (5.08-5.27)  3 (2- 4)  4 (4-9)  9.13 (8.46-9.99)  4 (2-9)  9 (2-9)  3.44 (2.42-4.16)  3 (2-4) | 3.24 (3.01-3.41)  5 (4-12)  6 (4-9)  6.2 (5.59-6.22)  2.5 (2.5-2.5)  5.14 (5.12-5.15)  5 (2- 12)  12 (9-12)  7.70 (7.60-8.29)  9 (9-9)  9 (9-9)  3.17 (2.74-3.24)  4 (2-9) | **0.028**  **0.017**  0.150  0.100  **0.021**  0.523  0.088  **0.045**  0.076  0.142  0.498  0.119  0.103 |
| ***SEROLOGY***  **VNT**  •First day positive  •First Day protective titre  •Day peaks  •Peak value (log_10_)  •Response time  **NSP**  •First day positive  •Response time  **Interferon γ**  •First day detected  •AUC (log_10_)  •Day peak  •Peak value (µg/mL)  •Response time  **Type I/III IFN**  •AUC (log_10_)  •Day peak  •Peak value (iu/mL)  •Response time | 4 (2 -6)  6 (4-6)  22 (14-30)  3.15 (3.15-3.15)  2 (0-4)  8 (6-8)  6 (4-6)  2 (2-2)  3.71 (3.17-3.99)  5 (2-6)  4.97 (3.91-5.48)  0 (0-0)  2.65 (1.96-2.79)  2 (2-4)  3.17 (1.24-3.65)  0 (0-0) | 6 (6-6)  6 (6-8)  11 (11-14)  3.15 (3.15-3.15)  4 (4-4)  8 (6-11)  6 (4-9)  2 (2-2)  3.90 (3.84-4.18)  4 (2-8)  5.09 (4.04-11.47)  0 (0-0)  2.69 (2.47-3.02)  2 (2-2)  2.93 (2.54-5.24)  0 (0-0) | 6 (2-6)  6 (6-8)  12.5 (8-14)  3.15 (3.15-3.15)  4 (0-4)  8 (6-8)  6 (4-6)  2 (2-2)  3.73 (3.47-4.45)  4 (2-14)  4.48 (4.04-5.22)  0 (0-0)  2.15 (1.49-2.61)  4 (2-6)  1.41 (0.66-3.83)  0 (0-0) | 0.134  0.294  0.063  -  0.134  0.829  0.829  -  0.276  0.995  0.608  -  0.219  0.238  0.397  - | 4 (2-6)  6 (6-9)  12 (9-12)  3.15 (3.15-3.15)  2 (0-4)  10.5 (9-12)  8.5 (7-10)  2 (2-2)  3.69 (6.66-3.76)  4 (4-4)  6.52 (6.13-7.86)  0 (0-0)  2.63 (2.14-2.82)  5 (4-6)  3.03 (1.38-5.07)  2 (0-2) | 9 (6-12)  10.5 (9-12)  12 (12-28)  3.15 (3.01-3.15)  6 (4-8)  10.5 (9-12)  7.5 (5-10)  2 (2-2)  3.83 (3.51-4.49)  4 (2-4)  6.95 (3.67-23.09)  0 (-2- 0)  2.42 (2.32-2.51)  6 (4-6)  2.27 (1.79-2.90)  0 (-2- 0) | 9 (6-12)  9 (9-12)  12 (12-28)  2.78 (2.25-3.15)  7 (2-8)  12 (12 – 28)  10 (8-24)  2 (2-2)  3.75 (3.74-3.85)  4(4-6)  4.98 (4.72-5.81)  -2 (-2 - 0)  1.77 (1.73-2.66)  4 (0-6)  1.97 (1.88-2.44)  1 (0 2) | **0.021**  **0.050**  c0.287  0.070  0.106  0.187  0.287  -  0.406  0.233  0.176  0.364  0.356  0.435  0.749  0.094 |
| ***HEMATOTOGY***  **HAPTOGLOBULIN**  •First day positive  •AUC (log_10_)  •Day peaks  •Peak value (ng/mL)  •Response time  **SAA**  •First day detected  •AUC (log_10_)  •Day peaks  •Peak value (ng/mL)  •Response time | 2 (2-2)  15.53 (15.46-15.58)  8 (4-11)  641082 (509902-671178)  0 (0-0)  2 (2-2)  11.35 (11.12-11.46)  4 (2-6)  12053 (10974-12822)  0 (0-0) | 2 (2-2)  16.01 (15.99-16.06)  11 (2-11)  666807 (643331-687700)  0 (0-0)  2 (2-2)  11.56 (10.83-11.75)  4 (4-6)  12364 (7711-15000)  0 (0-0) | 2 (2-2)  15.23 (14.66-15.66)  6 (4-11)  596071 (293986-660863)  0 (0-0)  2 (2-2)  11.19 (10.95-11.49)  5 (2-6)  12044 (10792-13825)  0 (0-0) | **-**  **0.018**  0.621  0.174  -  -  0.499  0.852  0.926  - | 4 (2-4)  15.90 (15.47-16.12)  9 (9-12)  654360 (537771-671676)  2 (0-2)  2 (2-2)  11.46 (10.90-12.61)  6 (4-28)  14532 (9646-14883)  0 (0-0) | 4 (2-6)  15.71 (15.40-16.0)  12 (6-12)  629915 (560970-659085)  2 (-2-2)  3 (2-6)  11.25 (11.04-11.72)  6 (6-6)  13974 (13159-15000)  1 (-2 - 2) | 4 (2-12)  13.13 (10.21-15.64)  9 (6-9)  154080 (3784-635514)  0.5 (-2 - 4)  2 (2-2)  11.72 (11.30-11.74)  6 (6-9)  14536 (13936-15000)  -2 (-2 0) | 0.903  0.138  0.294  0.161  0.962  -  0.446  0.732  0.722  0.219 |
