# Supplementary material for: Viral dynamics and immune responses to foot-and-mouth disease virus in African buffalo (Syncerus caffer)
Source: Vet Res. 2022 Aug 4;53:63. doi: 10.1186/s13567-022-01076-3 (PMC9351118; doi:10.1186/s13567-022-01076-3)
Supplement: Supplementary file 4 — Additional file 4. Median values (minimum–maximum) and the Kruskal–Wallis statistics of virus load, serology and hematology values stratified by method of infection (needle infected vs contact). [file 13567_2022_1076_MOESM4_ESM.docx]

| **Parameter** | **Median (min-max)** | | ***P* value** |
| --- | --- | --- | --- |
|  | **Needle infected** | **Contact** |  |
| ***VIROLOGY***  **Virus load in serum**  •AUC (log_10_)  •Day viremia starts  •Day viremia peaks  •Peak value  •Duration (days)  **Virus load in tonsils**  •AUC (log_10_)  •First day detected  •Day peaks  •Peak value  **Nasal swab**  •First day detected  •Day peaks  •Peak value  **Day virus first detected** | 3.22 (2.92-3.61)  2 (2-2)  2 (2-4)  6.35 (5.20-7.67)  4 (4-6)  5.36 (5.11-5.49)  2 (2-2)  4 (2-6)  9.34 (7.99-9.98)  2 (2-11)  4 (2-11)  3.42 (0-6.57)  2 (2-2) | 3.24 (2.00-3.53)  2 (2-12)  4 (4-9)  5.59 (2.50-8.39)  4.5 (2.5-6.5)  5.15 (4.82-5.37)  4 (2 – 12)  6 (4-12)  8.92 (7.60-9.99)  6.5 (2-9)  9 (2-9)  3.34 (2.42-6.47)  2 (2-9) | 0.389  **0.002**  **<0.001**  0.085  0.613  **0.004**  0.057  0.064  0.103  0.369  0.670  0.728  0.014 |
| ***SEROLOGY***  **VNT**  •First day positive  •First Day protective titre  •Day peaks  •Peak value (log10)  •Response time  **NSP**  •First day positive  •Response time  **Interferon γ**  •First day detected  •AUC (log_10_)  •Day peak  •Peak value (µg/mL)  •Response time  • Duration (days)  **Type I/III IFN**  •AUC (log_10_)  •Day peak  •Peak value (iu/mL)  •Response time  •Duration (days) | 4 (2-4)  6 (4-8)  14 (8-30)  3.15 (3.15-3.15)  4 (0-4)  8 (6-11)  6 (4-9)  2 (2-2)  3.83 (3.17-4.45)  5 (2-14)  4.90 (3.91-11.47)  0 (0-0)  14 (6-14)  2.58 (1.49-3.02)  2 (2-6)  2.93 (0.66-5.24)  0 (0-0)  6 (6-6) | 6 (2 – 12)  9 (6-12)  12 (9-28)  3.15 (2.25-3.15)  4 (0-8)  12 (9-28)  8 (5-24)  2 (2-2)  3.74 (3.51-4.49)  4 (2-6)  6.13 (3.67-23.09)  0 (-2 0)  9 (6-14)  2.47 (1.73-2.82)  6 (0-6)  2.18 (1.38-5.07)  0 (-2 2)  6 (0-9) | 0.062  **0.002**  0.751  **0.033**  0.132  **<0.001**  **0.001**  -  0.623  0.696  **0.036**  **0.031**  0.052  0.355  **0.013**  0.580  0.152  0.528 |
| ***HEMATOTOGY***  **HAPTOGLOBULIN**  •First day positive  •AUC (log_10_)  •Day peaks  •Peak value (ng/mL)  •Response time  **SAA**  •First day detected  •AUC (log_10_)  •Day peaks  •Peak value (ng/mL)  •Response time  •Duration (days) | 2 (2-2)  15.56 (14.66-16.06)  8 (2-11)  644713 (293986-687700)  0(0-0)  2(2-2)  11.35 (10.83-11.75)  4 (2-6)  12154 (7711-15000)  0 (0-0)  11 (8-14) | 4 (2 – 12)  15.64 (10.21-16.12)  9 (6-12)  635514 (3784-671676)  2 (-2-4)  2 (2-6)  11.38 (10.90-12.61)  6 (4-28)  14217(9646-15000)  0 (-2 2)  12 (9-28) | **<0.001**  0.902  0.118  0.325  0.057  -  0.460  **0.004**  **0.007**  0.622  **0.002** |
