# Supplementary material for: Viral dynamics and immune responses to foot-and-mouth disease virus in African buffalo (Syncerus caffer)
Source: Vet Res. 2022 Aug 4;53:63. doi: 10.1186/s13567-022-01076-3 (PMC9351118; doi:10.1186/s13567-022-01076-3)
Supplement: Supplementary file 6 — Additional file 6. RT-qPCR data expressed as Log10 FMDV GCN/mL from tonsil swabs at day 30 of the study using the 3D and SAT specific primers and probe. [file 13567_2022_1076_MOESM6_ESM.docx]

| Log_10_ FMDV GCN/mL from tonsil swab at 30 days of the study | | | | | | |
| --- | --- | --- | --- | --- | --- | --- |
|  |  | ID buffalo | 3D | SAT1 | SAT2 | SAT3 |
| SAT1 | SAT1 NI | 7 | 8.07 | 8.41 | 0.00 | 0.00 |
|  |  | 10 | 8.05 | 8.15 | 0.00 | 0.00 |
|  |  | 11 | 8.38 | 8.80 | 0.00 | 0.00 |
|  |  | 13 | 7.47 | 7.82 | 0.00 | 0.00 |
|  | SAT1 Co | 2 |  |  |  |  |
|  |  | 4 | 7.29 | 7.72 | 0.00 | 0.00 |
|  |  | 19 | 7.15 | 7.96 | 0.00 | 0.00 |
|  |  | 33 | 8.30 | 8.55 | 0.00 | 0.00 |
| SAT2 | SAT2 NI | 8 | 7.68 | 0.00 | 7.11 | 0.00 |
|  |  | 20 | 5.76 | 0.00 | 5.57 | 0.00 |
|  |  | 28 | 4.85 | 0.00 | 5.22 | 0.00 |
|  |  | 32 | 7.96 | 0.00 | 7.85 | 0.00 |
|  | SAT2 Co | 5 | 5.86 | 0.00 | 5.23 | 0.00 |
|  |  | 9 | 6.25 | 0.00 | 6.05 | 0.00 |
|  |  | 22 | 5.89 | 0.00 | 0.00 | 0.00 |
|  |  | 29 | 5.50 | 0.00 | 5.31 | 0.00 |
| SAT3 | SAT3 NI | 26 | 7.27 | 0.00 | 0.00 | 7.94 |
|  |  | 27 | 6.00 | 0.00 | 0.00 | 6.75 |
|  |  | 34 | 6.51 | 0.00 | 0.00 | 6.77 |
|  |  | 35 | 6.19 | 0.00 | 0.00 | 8.30 |
|  | SAT3 Co | 12 | 5.68 | 0.00 | 0.00 | 6.15 |
|  |  | 15 | 5.63 | 0.00 | 0.00 | 6.49 |
|  |  | 16 | 7.57 | 0.00 | 0.00 | 8.30 |
|  |  | 17 | 5.67 | 0.00 | 0.00 | 6.55 |
