# Supplementary material for: Viral dynamics and immune responses to foot-and-mouth disease virus in African buffalo (Syncerus caffer)
Source: Vet Res. 2022 Aug 4;53:63. doi: 10.1186/s13567-022-01076-3 (PMC9351118; doi:10.1186/s13567-022-01076-3)
Supplement: Supplementary file 6 [file 13567_2022_1076_MOESM5_ESM.docx]

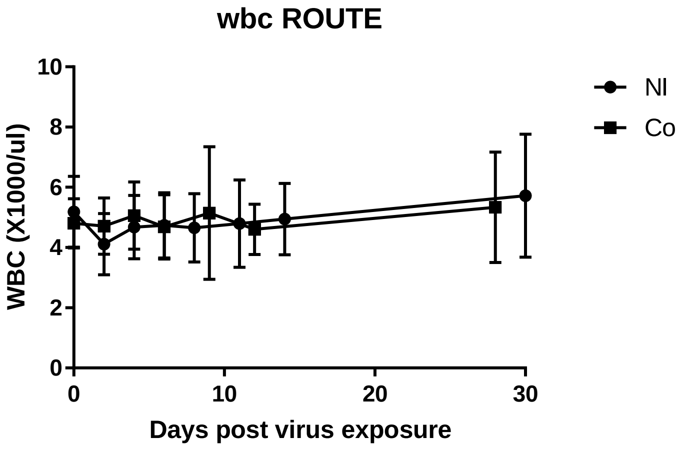
wbc ROUTE
WBC (X1000/ul)
Days post virus exposure
NI
Co
0
2
4
6
8
10
0
10
20
30
